# Tick Thioester-Containing Proteins and Phagocytosis Do Not Affect Transmission of *Borrelia afzelii* from the Competent Vector *Ixodes ricinus*

**DOI:** 10.3389/fcimb.2017.00073

**Published:** 2017-03-16

**Authors:** Veronika Urbanová, Ondřej Hajdušek, Helena Hönig Mondeková, Radek Šíma, Petr Kopáček

**Affiliations:** Biology Centre of the Czech Academy of Sciences, Institute of ParasitologyCeske Budejovice, Czechia

**Keywords:** *Borrelia*, complement, *Ixodes*, phagocytosis, thioester-containing proteins

## Abstract

The present concept of the transmission of Lyme disease from *Borrelia*-infected *Ixodes* sp. ticks to the naïve host assumes that a low number of spirochetes that manage to penetrate the midgut epithelium migrate through the hemocoel to the salivary glands and subsequently infect the host with the aid of immunomodulatory compounds present in tick saliva. Therefore, humoral and/or cellular immune reactions within the tick hemocoel may play an important role in tick competence to act as a vector for borreliosis. To test this hypothesis we have examined complement-like reactions in the hemolymph of the hard tick *Ixodes ricinus* against *Borrelia afzelii* (the most common vector and causative agent of Lyme disease in Europe). We demonstrate that *I. ricinus* hemolymph does not exhibit borreliacidal effects comparable to complement-mediated lysis of bovine sera. However, after injection of *B. afzelii* into the tick hemocoel, the spirochetes were efficiently phagocytosed by tick hemocytes and this cellular defense was completely eliminated by pre-injection of latex beads. As tick thioester-containing proteins (T-TEPs) are components of the tick complement system, we performed RNAi-mediated silencing of all nine genes encoding individual T-TEPs followed by *in vitro* phagocytosis assays. Silencing of two molecules related to the C3 complement component (*Ir*C3-2 and *Ir*C3-3) significantly suppressed phagocytosis of *B. afzelii*, while knockdown of *Ir*Tep (insect type TEP) led to its stimulation. However, RNAi-mediated silencing of T-TEPs or elimination of phagocytosis by injection of latex beads in *B. afzelii*-infected *I. ricinus* nymphs had no obvious impact on the transmission of spirochetes to naïve mice, as determined by *B. afzelii* infection of murine tissues following tick infestation. This result supports the concept that *Borrelia* spirochetes are capable of avoiding complement-related reactions within the hemocoel of ticks competent to transmit Lyme disease.

## Introduction

Ticks are obligatory blood-feeders capable of transmitting a wide variety of pathogens including viruses, bacteria, protozoa, fungi, or nematodes to their vertebrate hosts (Jongejan and Uilenberg, 2004; De La Fuente et al., 2008). Yet, the vector competence of different tick species is quite specific and is determined by the ability of any particular pathogen to overcome several barriers on its route from the infected tick to the naïve host, including defense mechanisms within the tick midgut, hemocoel, and salivary glands; for review see (Hajdusek et al., 2013). One of the most thoroughly investigated tick-borne diseases of humans is Lyme disease, caused by spirochetes of the genus *Borrelia* (Burgdorfer et al., 1982; Stanek et al., 2012) transmitted mainly by *Ixodes scapularis* in the USA and *Ixodes ricinus* in Europe (Piesman and Gern, 2004; Radolf et al., 2012). Several tick molecules have been described to play important roles in the *Borrelia* transmission cycle; reviewed in Hajdusek et al. (2013) and Kung et al. (2013): Glutathione peroxidase Salp 25D facilitates spirochete acquisition from the infected host (Narasimhan et al., 2007); TROSPA, tick receptor for the outer surface protein A, plays a role in *Borrelia* long-term persistence within the tick gut (Pal et al., 2004); tre31 that binds to another outer *Borrelia* surface lipoprotein, BBE 31, allows crossing of the midgut barrier (Zhang et al., 2011). Salivary proteins Salp15 (Ramamoorthi et al., 2005), tick histamine release factors (Dai et al., 2010), and tick salivary lectin pathway inhibitor (Schuijt et al., 2011) protect *Borrelia* at the tick-host interface via modulation of the host immune response. In addition to tick molecules, transmission of spirochetes is also aided by proteins originating from the blood meal, such as host plasminogen that is bound and activated on the spirochete surface, facilitating *Borrelia* migration through the tick and dissemination in the host (Coleman et al., 1997).

A previous study suggested that the difference in capacity to transmit *Borrelia burgdorferi* between *I. scapularis* (competent tick) and *Dermacentor variabilis* (refractory tick) is likely to be due to higher borreliacidal and phagocytic activities in the hemolymph of *D. variabilis* (Johns et al., 2001). Despite this important observation, our current knowledge on tick-*Borrelia* interactions in the hemocoel of *Ixodes* sp. ticks is rather limited. Phagocytosis of *B. burgdorferi* by *I. scapularis* hemocytes upon spirochete penetration from the midgut to the hemocoel has been described in several studies (Coleman et al., 1997; Dunham-Ems et al., 2009) but whether this cellular defense reaction plays any role in *Borrelia* transmission in a competent vector remains unclear.

In vertebrate animals, including mammalian, avian or reptile hosts, the decisive role in susceptibility or resistance to infection by a certain genospecies of *B. burgdorferi* sensu lato complex, is most likely played by the serum complement system (Kurtenbach et al., 1998, 2002; Kuo et al., 2000; Bhide et al., 2005; De Taeye et al., 2013). A borreliacidal effect on spirochetes was reported to be exerted by the alternative pathway of mammalian complement (Kurtenbach et al., 1998; Kuo et al., 2000). Since ticks possess a primitive complement system, comprising thioester-containing proteins (TEPs), ficolin-like lectins or putative C3-convertases (Kopacek et al., 2012), we primarily asked whether or not tick complement plays a role in the competence of ticks to act as a vector for Lyme disease. In this study we focused on molecules of the TEP family, which, in *Ixodes* sp. ticks, involves representatives of four major classes of TEPs known in invertebrates: (i) three proteins related to C3-complement component; (ii) three different α_2_-macroglobulins; (iii) one insect-type TEP, and (iv) two macroglobulin-complement-related (MCR) molecules (Buresova et al., 2011; Urbanova et al., 2015). Using RNAi-mediated silencing of individual genes encoding tick TEPs (T-TEPs), followed by *in vitro* phagocytosis assays, we have previously demonstrated that different T-TEPs are involved in phagocytosis of different model microbes (Gram-negative *Chryseobacterium indologenes, Escherichia coli* and yeast *Candida albicans*) by tick hemocytes (Buresova et al., 2009, 2011; Urbanova et al., 2015). However, a similar study focusing on *Borrelia* sp. spirochetes has not been performed yet, mainly due to the lack of a reliable phagocytic assay for this tick-borne pathogen. To overcome this problem, we have implemented a phagocytic assay for *Borrelia* sp. that exploits dual labeling, making it possible to clearly distinguish free and/or attached spirochetes from those being engulfed by tick hemocytes. Phagocytosis of different microbes by invertebrate hemocytes could be efficiently blocked by intra-hemocoelic injection of inert particles such as latex or polystyrene beads, as previously demonstrated for the fruit fly *Drosophila melanogaster* (Nehme et al., 2011) or the tick *I. scapularis* (Liu et al., 2011). With this experimental background, and using a recently established laboratory transmission model for *B. afzelii* (the most important agent of Lyme disease in Europe), we have examined the role of T-TEPs in phagocytosis of spirochetes by tick hemocytes and addressed the question of whether or not this cellular defense plays a role in spirochete transmission from *Borrelia-*infected *I. ricinus* nymphs to naïve mice. Our results collectively demonstrate that complement-like molecules are involved in tick phagocytic responses to *B. afzelii*, but do not prevent transmission of *B. afzelii*. These findings add to our understanding of the competence of this tick species to act as a vector for Lyme disease.

## Materials and methods

### Biological material

Adult females and males of *I. ricinus* were collected by flagging in woodlands around České Budějovice, the Czech Republic. All developmental stages (eggs, larvae, nymphs, and adults) were maintained in wet chambers with a humidity of about 95%, temperature 24°C and day/night period set to 15/9 h. Females were fed naturally on laboratory guinea pigs. The larvae were fed on guinea pigs, allowed to molt to nymphs and, after 4–6 weeks, further fed on guinea pigs or rabbits. The nymphs (pathogen free or infected with *B. afzelii* CB 43) and adult females (pathogen free) were used for experiments described below. All laboratory animals were treated in accordance with the Animal Protection Laws of the Czech Republic No. 246/1992 Sb., ethics approval No. 095/2012.

### Borreliacidal assay

*B. afzelii* CB43 spirochetes were cultivated in BSK-H complete medium (Sigma-Aldrich) at 33°C for 5–7 days and for the assay, were diluted to a concentration of 5 × 10^6^ cells/ml. Hemolymph samples from 50 semi-engorged females (6th day of feeding) were collected into a glass capillary from the cut front leg, immediately cooled on ice and the collected pool was centrifuged at 300 × g for 10 min. The supernatant was transferred to a fresh tube and centrifuged at 9500 × g for 10 min. The hemocyte-free plasma so obtained was used for subsequent experiments. Bovine serum was prepared from manually defibrinated bovine blood as described previously (Perner et al., 2016) and inactivated bovine serum was prepared by heating at 56°C for 40 min.

The assay was performed in 96-well plates (Nunc) by adding 50 μl of *B. afzelii* (5 × 10^6^/ml) to 10 μl of tested sample (tick plasma, bovine serum, inactivated bovine serum, or BSK-H medium as a control), and incubated for 24 h in a wet chamber at 33°C. The number of live *B. afzelii* was determined using dark-field microscopy as follows. The *Borrelia* culture (3.5 μl) was transferred onto a microscope slide and covered with 18 × 18 mm coverslip. Spirochetes present in the view field were counted and the average number, calculated from 10 fields counts, was multiplied by the coefficient 3.9 × 10^5^ pre-determined for our microscope Olympus, model BX53 (total magnification—400x). The obtained results thus represent the number of *Borrelia* normalized for 1 ml culture volume.

### *In vivo* phagocytosis of *Borrelia afzelii* CB43

Pathogen free, adult *I. ricinus* females were fed naturally for 6 days on guinea pigs. Cultivated *B. afzelii* (5 × 10^4^ spirochetes per tick) were injected into the hemocoel of semi-engorged females in a volume of 138 nl by microinjection (microinjector Drummond). Hemolymph samples from individual ticks were collected at defined time points (0, 1, 3, and 6 h) after injection of spirochetes and mixed with 10 μl of L15-BOFES medium supplemented with 10% fetal calf serum (PAA Laboratories) on microscope slides. Cells were fixed with 4% formaldehyde in phosphate saline buffer (PBS) for 20 min and washed 3 times with PBS. Spirochetes were stained with primary rabbit anti-*B. burgdorferi* (Thermo Scientific) antibody (1:200 in PBS), on the slides and incubated on a horizontal shaker at room temperature (RT) for 1 h. After washing with PBS (3 times/5 min), slides were stained with fluorescently labeled goat anti-rabbit secondary antibody (Alexa 594) (Molecular Probes) diluted 1:500 in PBS and incubated for 1 h at RT. Hemocytes were then permeabilized using 1% Triton X-100 in PBS with 1% BSA (Bovine serum albumin, Sigma-Aldrich) overnight at 4°C. The next day, spirochetes were restained with primary antibody (rabbit anti-*B. burgdorferi*) diluted 1:200 in 0.1% Triton in PBS (PBS-Tx) for 1 h at room temperature, and washed 3 × 5 min with 0.1 % PBS-Tx. Spirochetes were then stained with anti-rabbit secondary antibody (Alexa 488) (Molecular Probes), diluted 1:500 in PBS-Tx for 1 h at RT. Cells were then washed with PBS-Tx and nuclei were counterstained with DAPI for 10 min. After mounting in DABCO (Sigma), the number of phagocytic hemocytes was counted using a 488/594 (FITC/TexasRed) dual filter and BX51 fluorescent microscope (Olympus). For each sample, 100 hemocytes were counted.

For the experiment with latex beads (LtxB), 138 nl of 2x diluted surfactant-free red CML latex beads, 0.3 μm diameter (Interfacial Dynamics Corp.) were injected into semi-engorged fed females and, after 2 h, ticks were injected with *B. afzelii* CB43 (5 × 10^4^). Hemolymph from individual ticks was collected 3 h after injection of *Borrelia* and phagocytic activity of hemocytes was analyzed (12 ticks for PBS control group and 15 ticks for latex beads group) as described above. The phagocytic index was determined as the number of hemocytes with ingested *Borrelia* counted for a total 100 hemocytes in the microscopic field.

### Determination of hemocyte number after injection of latex beads

Unfed *I. ricinus* females (25 per group) were injected with 138 nl of 2x diluted LtxB or PBS (control) and allowed to rest for 1 day. After that, females were fed naturally on guinea pigs for 6 days and then hemolymph samples from individual females were collected on microscope slides. Cells were fixed with 4% formaldehyde in PBS for 20 min and washed 3 times with PBS. Hemocyte nuclei were counterstained with DAPI. Hemocytes were counted on the whole slide using a BX51 Olympus fluorescence microscope.

### RNAi-mediated silencing of tick TEPs linked with *in vitro* phagocytosis assay

dsRNAs of nine *t-teps* and *gfp* (control) were produced as described previously (Buresova et al., 2011). For each experiment, *t-teps*-specific dsRNA (0.5 μl; 3 μg/μl) was injected into the hemocoel of 25 unfed *I. ricinus* females through to the coxae using a microinjector (Drummond). Ticks were allowed to rest for 1 day and then fed for 6 days on guinea pigs. Hemolymph samples collected from 25 semi-engorged females were mixed with L15-BOFES medium supplemented with 10% fetal calf serum (PAA Laboratories) (Buresova et al., 2011; Urbanova et al., 2015). Hemocytes (4 × 10^4^) in a volume of 150 μl were transferred onto round microscope cover slips in a 24-well culture plate, and then 10 μl of *B. afzelii* CB43 (10^8^ cells/ml) were added and incubated for 2 h at 28°C. Cells were fixed with 4% formaldehyde in PBS for 20 min and washed 3 times with PBS. Spirochetes were detected by indirect immunofluorescence, using the method of double staining as described above. Phagocytosed spirochetes were counted using the BX51 Olympus fluorescence microscope. For each group, 100 hemocytes were counted on each of at least 14 slides. Relative phagocytosis was calculated in relation to the number of phagocytic hemocytes in the *gfp* dsRNA injected control group, taken as 100% for each respective experiment.

### Expression of genes encoding *I. ricinus* thioester-containing proteins in response to injection of *Borrelia* sp. spirochetes

Different species of *Borrelia* (*B. burgdorferi* NE5264, *B. burgdorferi* CB26, *B. garinii* MSLB, *B. afzelii* CB43) were cultivated in BSK-H complete medium (Sigma) at 33°C for 5–7 days. All *Borrelia* species were diluted in PBS to contain 10^4^ spirochetes in the injection dose. Unfed, pathogen free *I. ricinus* females were surface-sterilized by a subsequent immersion into 3% H_2_O_2_, 70% EtOH and sterile distilled water. A 69 nl volume of *Borrelia* suspension in sterile PBS, or BSK-H medium for controls, was injected into ticks using sterile glass capillaries and a microinjector (Drummond). After inoculation, ticks were allowed to rest for 12 h at room temperature, total RNA was extracted from the whole body homogenates using TRI reagent® (Sigma), treated with DNAse (Ambion), and the integrity of RNA was checked by agarose gel electrophoresis. Single-stranded cDNA was reverse-transcribed from 0.5 μg of total RNA using the Transcriptor High-Fidelity cDNA Synthesis Kit (Roche). The resulting cDNA preparations served as templates for subsequent expression analysis by quantitative real-time PCR (qPCR) using a LightCycler 480 (Roche) and SYBR green chemistry. Reaction conditions and sequences of qPCR T-TEPs forward and reverse primers have been published previously (Urbanova et al., 2015). Relative expression of *t-teps* was normalized to elongation factor 1 (*ef-1*) using the mathematical model of Pfaffl (Pfaffl, 2001). For each experimental group, five *I. ricinus* females were injected in three independent biological triplicates.

### *Borrelia* transmission

*B. afzelii* CB43 spirochetes were cultivated as described above. To prepare *Borrelia*-infected nymphs for the transmission experiment, C3H/HeN mice were injected intra-dermally with 10^5^ of *B. afzelii* spirochetes. After 4 weeks, pathogen-free larvae were fed on infected mice (~100 larvae per mouse) and after repletion were kept in wet chambers at 26°C until molting. The infected nymphs (50 per group) were injected with mixed or individual dsRNAs (3 μg/μl, 64.4 nl) specifically silencing the group of IrAMs (*iram-1,2,3)*, IrC3s (*irc3-1,2,3*), IrMCRs (*irmcr-1,2*), IrTep (*irtep*), *gfp* (control). For experiments with latex beads, 50 unfed *B. afzelii*-infected nymphs were micro-injected with LtxB (32 nl, 2x diluted) and 50 with sterile PBS as a control group. Following injection, nymphs were allowed to rest for 3 days, and then were fed until repletion on clean 6-weeks old C3H/HeN mice (10 nymphs per mouse, 5 mice per each experimental group) using plastic cylinders attached to the murine back. Infection of mice with *Borrelia* during the early phase following tick infestation was tested in ear tissue biopsies taken at 1 week intervals. After 4 weeks the mice were sacrificed and the *Borreli*a spirochetes were detected in the target tissues, namely the bladder and heart. The mice tissues were first tested for *Borrelia* positivity using sensitive PCR amplification of a 154 bp fragment of the *flagellin* gene. The PCR reactions contained 12.5 μl of FastStart PCR MasterMix (Roche), 4 μl of DNA extracted using Macherey-Nagel NucleoSpin®Tissue Kit (concentration in the range of 100–300 ng), 10 pmol of each primer FlaF1 (AAGCAAATTTAGGTGCTTTCCAA), FlaR1 (GCAATCATTGCCATTGCAGA) and PCR water up to 25 μl. The amplification program consisted of denaturation at 94°C for 10 min, then 40 cycles of: denaturation at 94°C for 30 s, annealing at 60°C for 30 s and elongation at 72°C for 40 s. The program was finished by final extension at 72°C for 7 min. PCR products were visualized on a 1.5% agarose gel. Positive tissues were further analyzed by qPCR using a LightCycler 480 (Roche). qPCR was performed in a 25 μl reaction volume containing 12.5 μl of FastStart Universal Probe Master (Rox) (Roche), 5 μl of purified DNA, 10 pmol of each primer, FlaF1, FlaR1, and 5 pmol of TaqMan probe, FlaProbe1 (FAM-TGCTACAACCTCATCTGTCATTGTAGCATCTTTTATTTG-BHQ1) (Schwaiger et al., 2001). The remaining reaction volume was adjusted with PCR water. The qPCR program consisted of denaturation at 95°C for 10 min, followed by 50 cycles of: denaturation at 95°C for 15 s, annealing plus elongation at 60°C for 1 min. The number of spirochetes in tissues was normalized to the number of murine genomes as described previously (Dai et al., 2009).

### Statistical analysis

The appropriate statistical analyses (non-parametric Kruskal-Wallis test, non-parametric Mann-Whitney test or un-paired *t*-test) were selected for the specific data-sets and specified in the legends to the corresponding figures. All statistics was performed using GraphPadPrism (version 6.00 for Windows, GraphPad Software, San Diego, CA, USA). A *P*-value of < 0.05 was considered to be statistically significant.

## Results

### *Ixodes ricinus* plasma did not exhibit complement-mediated borreliacidal activity similar to bovine serum

**Figure 1:**
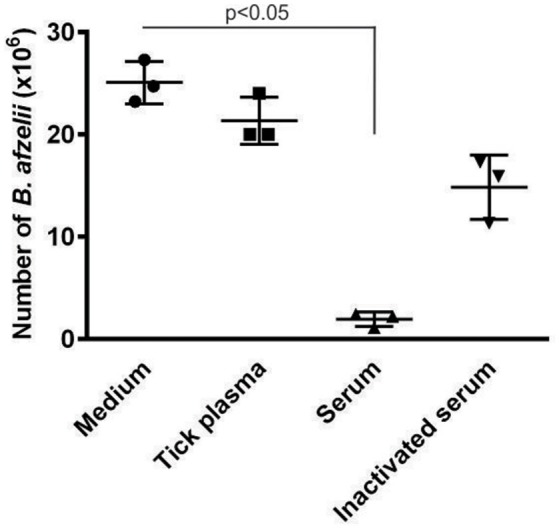
**Plasma of *I. ricinus* exhibits no borreliacidal activity against *B. afzelii* spirochetes**. Spirochetes were incubated for 24 h in the presence of BSK-H medium (negative control), *I. ricinus* hemocyte-free plasma, bovine serum or heat-inactivated bovine serum. Numbers of live *Borrelia* were normalized to 1 ml volume. This result demonstrates that complement-like molecules in tick hemolymph do not exert borreliacidal effects similar to that of the complement system present in bovine serum. Data were analyzed by non-parametric Kruskal-Wallis test.

Complement systems in sera of various vertebrate animals exhibit different borreliacidal effects against different *Borrelia* genospecies (Kurtenbach et al., 2002; Bhide et al., 2005; Ticha et al., 2016). Because *Ixodes* sp. ticks possess a primordial complement system involving three molecules related to the C3 complement component and putative convertases (Buresova et al., 2011; Kopacek et al., 2012; Urbanova et al., 2014) we tested for possible effects of tick hemolymph on *Borrelia* viability. Cultivated *B. afzelii* spirochete*s* were incubated with tick cell-free plasma, bovine serum, heat-inactivated bovine serum or BSK-H medium and tested for *Borrelia* survival after 24 h of incubation *in vitro*. Incubation of bovine serum with *B. afzelii* resulted in spirochete immobilization, lysis and cluster formation. This effect was markedly reduced by heat inactivation of serum complement. In contrast, no borreliacidal effect was observed upon incubation of spirochetes with tick plasma or BSK-H medium used as a negative control (Figure 1).

### Tick hemocytes were capable of phagocytosing *Borrelia afzelii* injected into the hemocoel

**Figure 2:**
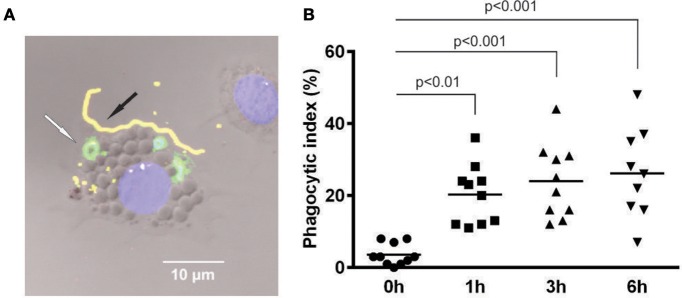
*****B. afzelii*** spirochetes are actively phagocytosed by tick hemocytes upon inoculation into the tick hemocoel. (A)** Double immunostaining of phagocytic *I. ricinus* hemocytes distinguished between ingested and attached *B. afzelii*. Green—ingested *Borrelia* inside the hemocyte (white arrow); Yellow—attached *Borrelia* outside the hemocyte (black arrow); Blue—nucleus of hemocyte-stained with DAPI; Nomarski contrast. **(B)** Semi-engorged females were injected with 5 × 10^4^ of *B. afzelii* CB43. Hemolymph samples from different time points after injection were collected from individual ticks (10 ticks per time point) and the phagocytic index was determined as the number of hemocytes with ingested *Borrelia* per 100 hemocytes in the microscope field. Data were analyzed by non-parametric Kruskal-Wallis test.

As no humoral reaction against *B. afzelii* was observed in tick plasma, we further investigated phagocytic activity of tick hemocytes against spirochetes injected into the tick hemocoel. *B. afzelii* spirochetes were injected into semi-engorged females, and phagocytosis was examined in hemolymph collected at different time intervals post injection. In order to distinguish between *Borrelia* spirochetes that were ingested by tick hemocytes from attached or free spirochetes, a dual-labeling assay was exploited (Figure 2A). We observed that spirochetes were phagocytosed immediately after injection and a phagocytic rate of about 25% was reached after 1 h; this was maintained for at least 6 h (Figure 2B).

### Phagocytosis of *B. afzelii* was eliminated by injection of latex beads

**Figure 3:**
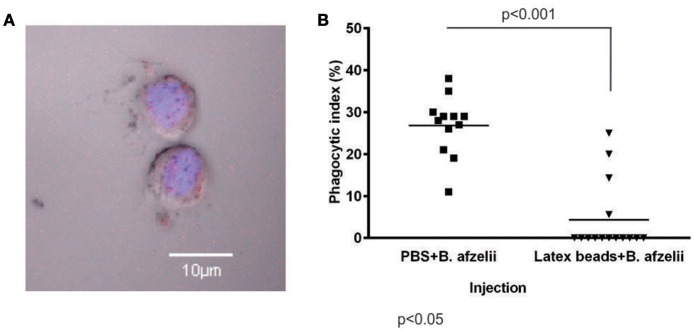
**Phagocytosis of *B. afzelii* by tick hemocytes is eliminated by pre-injection of latex beads into the hemocoel. (A)** Tick hemocyte with ingested latex beads. Red—latex beads; Blue—nucleus of hemocytes-stained with DAPI; Nomarski contrast. **(B)** Pre-injection of latex beads (138 nl, 2x diluted) into the hemocoel significantly reduced phagocytosis of *B. afzelii* CB43 injected 2 h later. Hemolymph was collected from individual ticks (12 ticks for PBS and 15 ticks for latex beads) 3 h after injection of *Borrelia* and hemocytes were examined for phagocytosis of spirochetes. The phagocytic index was determined as the number of hemocytes with ingested *Borrelia* per 100 hemocytes in the microscope field. The number of hemocytes did not differ between experimental groups. Data were analyzed by non-parametric Mann-Whitney test.

Following the evidence that injection of latex or polystyrene beads suppresses phagocytosis in fruit fly (Nehme et al., 2011) or ticks (Liu et al., 2011), we performed an experiment whereby LtxB was injected into the hemocoel of semi-engorged *I. ricinus* females 2 h prior to injection of *B. afzelii*. Phagocytosis was evaluated as described above, 3 h after spirochete injection (Figure 3A). Pre-injection of LtxB resulted in a significant reduction in spirochete phagocytosis, where the phagocytic index decreased from 27% for the PBS control to 4% for LtxB pre-injection, while the numbers of hemocytes 5 h after LtxB or PBS injections were the same (Figure 3B).

**Figure 4:**
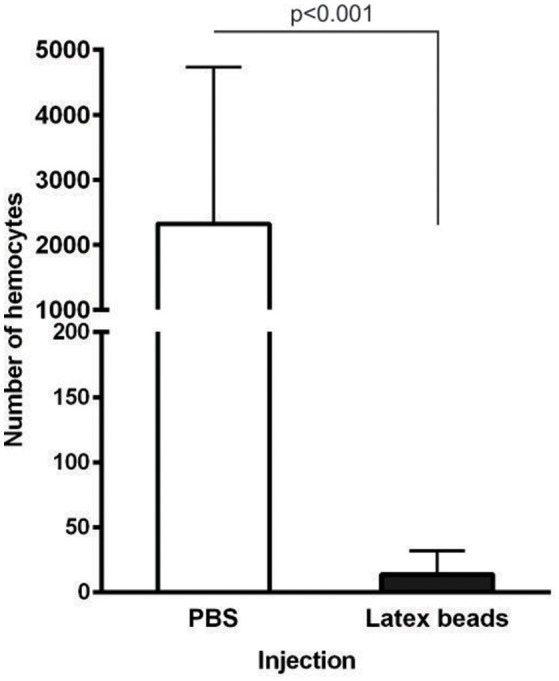
**Pre-injection of latex beads into unfed female *I. ricinus* results in hemocyte clearance from the tick hemocoel during feeding**. Unfed *I. ricinus* females were injected with LtxB (7 and 9 ticks) or sterile PBS (6 and 6 ticks) in two independent biological experiments. The females were allowed to feed naturally for 6 days and the hemolymph was collected from individual semi-engorged ticks. Hemocytes were counted on the whole slide. Data were analyzed by unpaired *t*-test. The error bars represent standard deviations.

Intriguingly, injection of LtxB into the hemocoel of unfed females that were further allowed to feed naturally for 6 days resulted in almost complete clearance of hemocytes from tick hemolymph compared to ticks pre-injected with PBS as a control (Figure 4). Despite this striking phenotype, the ticks were capable of feeding with no obvious impact on their fitness or fecundity.

### Function of tick TEPs in phagocytosis of *B. afzelii*

**Figure 5:**
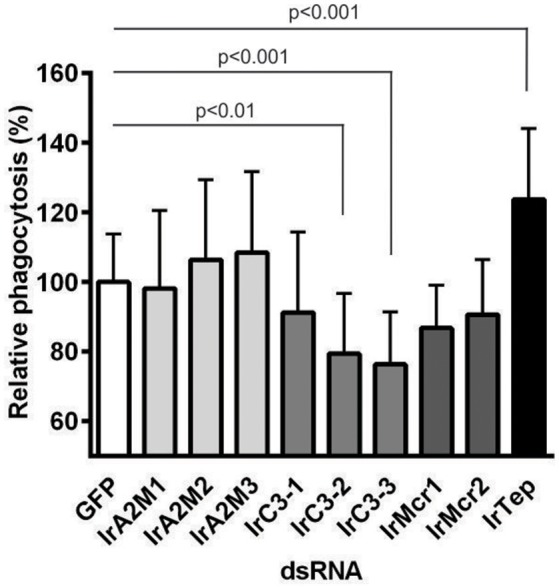
**The role of tick thioester-containing proteins in *Borrelia* phagocytosis**. Groups of 25 unfed *I. ricinus* females were injected with individual *t-teps* dsRNA or *gfp* dsRNA for control. Hemolymph collected and pooled from semi-engorged ticks was incubated with *B. afzelii* CB43 spirochetes *in vitro*. The number of phagocytic hemocytes upon silencing of individual *t-teps* was related to that obtained for the *gfp* dsRNA injected control group taken as 100% in the respective experiment. Average results (bars) and standard deviations (error bars) from three independent experiments are shown. Data were analyzed by Kruskal-Wallis test, followed by Dunn's multiple comparisons test.

In previous work, we demonstrated that different T-TEPs play non-redundant roles in the phagocytosis of Gram-negative bacteria (Buresova et al., 2011) or the yeast *C. albicans* (Urbanova et al., 2015). A similar experimental setup combining RNAi-mediated silencing of individual T-TEPs followed by an *in vitro* phagocytosis assay was used to identify T-TEPs playing a role in the phagocytosis of *Borrelia* spirochetes by tick hemocytes. Unfed *I. ricinus* females were injected with gene-specific *t-tep*s dsRNA or *gfp* dsRNA as a negative control. Ticks were allowed to feed naturally for 6 days, then the hemolymph was collected from semi-engorged females and used for an *in vitro* phagocytosis assay based on *B. afzelii* double immunostaining. Out of nine T-TEPs tested, only silencing of *irc3-2* and *irc3*-*3* significantly decreased phagocytosis of *B. afzelii*. In contrast, knockdown (KD) of the insect-type *irtep* led to a surprising increase in phagocytosis of spirochetes, by about 20% compared to the GFP control (Figure 5).

### Expression response of genes encoding *I. ricinus* thioester-containing proteins to injection of *Borrelia* sp. spirochetes

**Figure 6:**
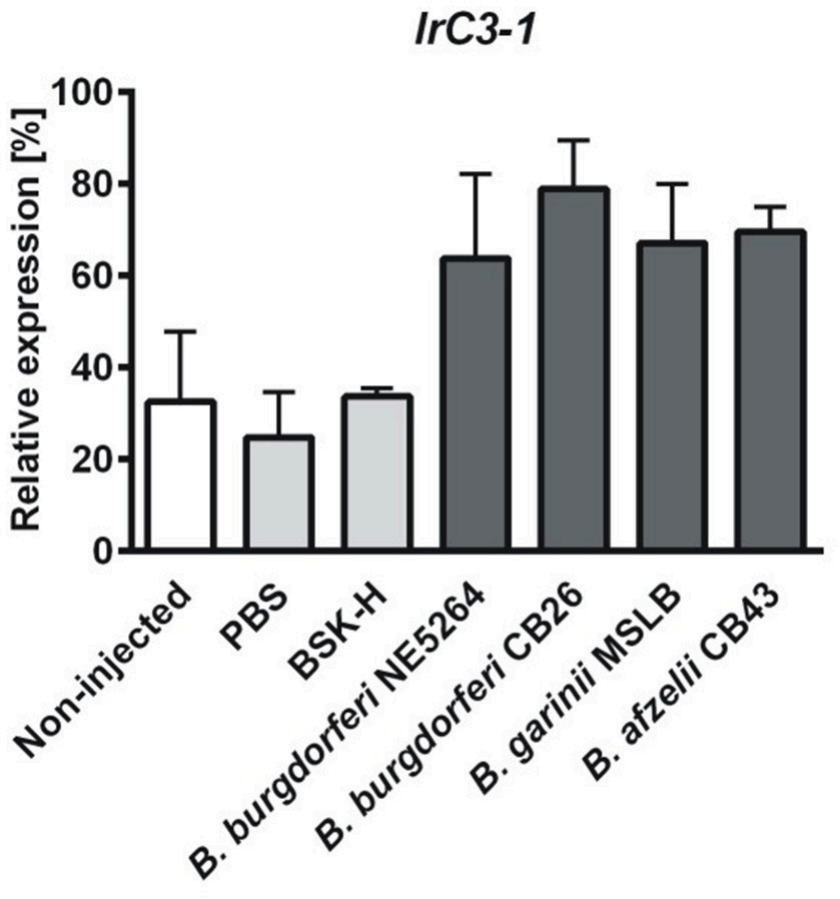
**Injection of *Borrelia* sp. affects expression of *irc3-1 gene***. Adult unfed *I. ricinus* females were injected with four different species of *Borrelia burgdorferi* s.l. complex (*B. burgdorferi* NE5264, *B. burgdorferi* CB26, *B. garinii* MSLB and *B. afzelii* CB43) or with sterile PBS, BSK-H medium as aseptic injection control and mock, respectively. Total RNA was isolated from the whole body homogenates, 12 h after inoculation, and transcribed into cDNA. Expression is shown in relation to *elongation factor-1* as a housekeeping gene. Expression of other *t-tep* genes were not responsive to *Borrelia* sp. injection (Figure S1). The error bars represent standard errors from three independent biological replicates. Data were analyzed by non-parametric Kruskal-Wallis test.

We have previously shown that expression of genes encoding *I. ricinus* T-TEPs responds differentially to different model microbes (*E. coli, Micrococcus luteus, or C. albicans*) or to aseptic injury (Urbanova et al., 2015). Here we have analyzed the expression response of *t-teps* to injection of available species of the *B. burgdorferi* sensu lato complex. Surface-sterilized unfed *I. ricinus* females were injected with sterile PBS as an aseptic injection control (injury), four different cultivated *Borrelia* species, and BSK-H medium alone as a mock. Total RNA was isolated 12 h after injection from whole body homogenates and mRNA levels of the genes encoding *t-teps* were determined by qPCR. Gene *irc3-1* was the only *t-tep* for which, expression seemed to be up-regulated upon injection of the tested *Borrelia* genospecies (2–3 times, in relation to the PBS and BSK-H injection controls) (Figure 6). Expression of other *t-teps* did not change in response to any *Borrelia* species or injection injury (Figure S1).

### Changes in *Borrelia* phagocytosis in ticks had no effect on spirochete burden in murine tissues

**Figure 7:**
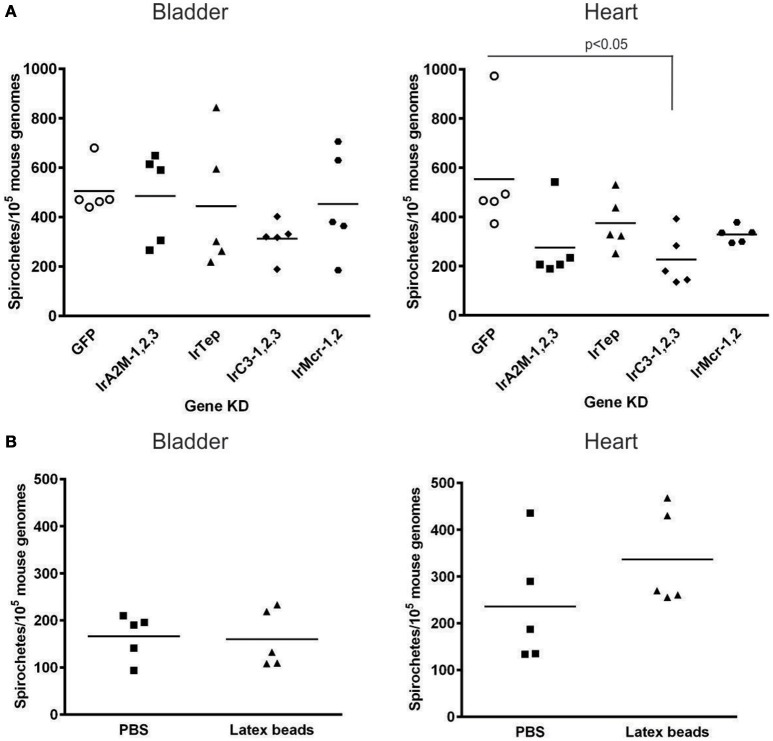
**Spirochete levels in murine bladder and heart biopsies in the 4th week after nymphal feeding. (A)** Infection of naïve mice with *B. afzelii* CB43 transmitted from naturally infected nymphs pre-injected with different group combinations of *t-tep* dsRNAs or *gfp* dsRNA as a control. **(B)** Infection of naïve mice with *B. afzelii* CB43 from naturally infected nymphs pre-injected with LtxB or PBS as a control. For each individual group (*t-teps, gfp*, LtxB, or PBS), five mice were infested with 10 nymphs. The number of *Borrelia* in the target tissues (bladder and heart) was determined by qPCR 4 weeks after infestation. The number of *Borrelia* was normalized to 10^5^ mouse genomes. **(A)** Data were analyzed by non-parametric Kruskal-Wallis test; **(B)** data were analyzed by unpaired *t*-test.

In order to examine whether or not immune reactions in the tick hemocoel affect *Borrelia* transmission to the host, we adapted the laboratory model for *Borrelia* transmission developed for *B. burgdorferi* sensu stricto and *I. scapularis* (Ramamoorthi et al., 2005; Dai et al., 2009) and applied it for *I. ricinus* nymphs infected with *B. afzelii* CB43 as described above. Groups of unfed, infected nymphs were injected with four combinations of dsRNAs, corresponding to the four classes of tick TEPs: (i) α_2_-macroglobulins (IrA_2_M-1,2,3); (ii) C3-complement component (IrC3-1,2,3); (iii) macroglobulin-complement-related (IrMcr-1,2), and (iv) insect-type IrTep, respectively. The efficiency of RNAi combinatorial KD was verified by qRT-PCR using cDNA prepared from a pool of five randomly selected nymphs. Additionally, we also tested whether the elimination of phagocytosis that followed pre-injection of LtxB (Figure 3B) affected transmission of *B. afzelii*. Mice infected with *Borrelia* during the early phase following tick infestation were tested in ear tissue biopsies taken at 1 week intervals. After 4 weeks, the mice were sacrificed and *Borrelia* spirochetes were detected in the bladder and heart (Table 1). The *Borrelia* numbers in ear biopsies fluctuated, ranging from a few to several thousand spirochetes, usually reaching a maxima from the 2nd to 3rd week following infestation and then gradually decreasing (Table S1). The spirochete burdens in bladders and hearts in the 4th week after infestation were relatively stable, in the range of several hundreds of spirochetes per murine genome (Figure 7). The only statistically significant decrease of *Borrelia* load compared to the GFP control was observed in mice heart upon group silencing of *irc3-1,2,3* genes. Based on these results we conclude that T-TEPS and phagocytosis do not substantially affect the competence of *I. ricinus* to act as a vector for Lyme disease.

**Table 1:** **PCR detection of *Borrelia afzelii* CB43^a^ in murine tissues after infestation with infected nymphs pre-injected with dsRNA or latex beads**.

**dsRNA KD/Injection**	**Ears 1st week**	**Ears 2nd week**	**Ears 3rd week**	**Ears 4th week**	**Bladder**	**Heart**
GFP	0/5	5/5	5/5	5/5	5/5	5/5
IrA_2_M-1,2,3	0/5	5/5	5/5	5/5	5/5	5/5
IrTep	0/5	5/5	5/5	5/5	5/5	5/5
IrC3-1,2,3	0/5	5/5	5/5	5/5	5/5	5/5
IrMcr-1,2	0/5	5/5	5/5	5/5	5/5	5/5
Latex beads	0/5	5/5	5/5	5/5	5/5	5/5

a *PCR detection based on amplification of flagellin B gene. Displayed are number of positive/number of examined mice*.

## Discussion

The concept of transmission of Lyme disease spirochetes from infected *Ixodes* sp. ticks to susceptible hosts via the salivary route, as proposed in the late eighties (Ribeiro et al., 1987), has been corroborated by a number of seminal studies published over the past three decades (De Silva and Fikrig, 1995; Coleman et al., 1997; Hojgaard et al., 2008; Dunham-Ems et al., 2009). Several tick proteins have been demonstrated to be involved in tick-*Borrelia* interactions and play roles in spirochete acquisition, midgut colonization, penetration of the midgut epithelium or shielding *Borrelia* against host immune and inflammatory responses at the tick-host interface (Pal et al., 2004; Ramamoorthi et al., 2005; Narasimhan et al., 2007; Dai et al., 2010; Schuijt et al., 2011; Zhang et al., 2011) or see (Hajdusek et al., 2013; Kung et al., 2013) for review. Except for the recently described antimicrobial peptide Dae2 (domesticated amidase effector), induced by tick GTPase (Chou et al., 2015; Smith et al., 2016), no other tick molecule has been described to limit *Borrelia* proliferation within a tick vector.

We have previously reported that ticks possess molecules related to components of the mammalian complement system (Buresova et al., 2011; Kopacek et al., 2012; Urbanova et al., 2015) and therefore we questioned whether tick cell-free plasma can exhibit lytic activity against *Borrelia*, as demonstrated for a variety of vertebrate animals (Kurtenbach et al., 1998, 2002; Kuo et al., 2000; Bhide et al., 2005; De Taeye et al., 2013; Ticha et al., 2016). The results shown in the Figure 1 clearly demonstrate that there is almost no effect of *I. ricinus* plasma against cultivated spirochetes compared to bovine serum, which is known to have strong complement-mediated borreliacidal activity (Kurtenbach et al., 1998; Bhide et al., 2005). This result is in accord with previous work demonstrating that plasma from *I. scapularis*, (the competent *Lyme disease* vector in the USA) had no borreliacidal activity (Johns et al., 2001). In contrast, plasma from the refractory *D. variabilis* substantially reduced the survival of incubated *Borrelia*. In addition, the authors also reported that clearance of spirochetes inoculated into the hemocoel of both species was much faster in *D. variabilis* compared to *I. scapularis* (Johns et al., 2001).

Among cellular reactions, phagocytosis is believed to play the most important defense role against microbial infections of arthropods, including ticks, and have been reported to suppress *Borrelia* numbers migrating through the tick hemolymph toward the salivary glands (Munderloh and Kurtti, 1995; Coleman et al., 1997; Dunham-Ems et al., 2009). Two different mechanisms, namely conventional and coiling phagocytosis, were reported for engulfment of *B. burgdorferi* by *I. ricinus* hemocytes (plasmatocytes and granulocytes of type II) similar to that of mammalian phagocytic cells (Rittig et al., 1996). Upon direct inoculation of cultivated *B. afzelii* into the hemocoel of *I. ricinus*, we observed an immediate and potent phagocytic activity of tick hemocytes, reaching a phagocytic index of about 25% (Figure 2). The phagocytic activity of tick hemocytes could be almost completely abolished by pre-injection of latex beads into the hemocoel (Figure 3).

Our *in vitro* phagocytosis assays following specific gene knockdown of individual *t-teps* demonstrated that phagocytosis of *B. afzelii* CB43 was significantly reduced upon silencing of *irc3-2* and *irc3-3* and increased upon knockdown of *irtep* (Figure 5). For comparison, phagocytosis of Gram-negative bacteria *C. indologenes* (pathogenic to ticks) (Buresova et al., 2006) is mediated mainly by IrA2M-1,2 and IrC3-3. We proposed that involvement of α_2_-macroglobulins in the cellular response against these bacteria was possible given the interaction of these macromolecular protease inhibitors with the potent Zn^2+^-dependent metalloprotease secreted by *C. indologenes* (Buresova et al., 2009). Phagocytosis of the model Gram-negative bacteria *E. coli* involves IrTep and IrC3-3 (Buresova et al., 2011), whereas for phagocytosis of the yeast *C. albicans*, the main role is carried out by IrC3-1 and IrMcr-2 (Urbanova et al., 2015). A similar non-redundant role of members of the TEP family has also been shown for the mosquito *Anopheles gambiae*, where silencing of *Ag*Tep1 and *Ag*Tep4 significantly inhibited phagocytosis of *E. coli* as well as the Gram-positive *S. aureus*, while RNAi silencing of mosquito *Ag*Tep3 only reduced phagocytosis of *E. coli* but not *S. aureus* (Moita et al., 2005). A study exploiting *D. melanogaster* S2 cells revealed that *Dm*Tep2, *Dm*Tep3 and *Dm*Tep6 were specifically required for phagocytosis of *E. coli, S. aureus*, and *C. albicans*, respectively (Stroschein-Stevenson et al., 2006). We also found that inoculation of different strains of the *B. burgdorferi* sensu lato complex up-regulated only *irc3-1* expression (Figure 6), while expression of other *t-teps* did not seem to be affected (Figure S1). A similar obvious up-regulation of *irc3-1* mRNA levels was observed upon injection of *C. albicans* (Urbanova et al., 2015), suggesting that the immune responses to *Borrelia* and yeast might be controlled by the same or related signaling pathways.

Earlier, it was elegantly demonstrated using fluorescent (GFP-expressing) *B. burgdorferi* that penetration of spirochetes from the midgut to the hemocoel was quite a rare event (Dunham-Ems et al., 2009), which agreed with other studies showing that the number of spirochetes that disseminate in tick hemolymph is very low compared to their massive presence in the tick midgut (Munderloh and Kurtti, 1995; Coleman et al., 1997; Zhang et al., 2011). The fact that the efficient phagocytic response of tick hemocytes is not capable of complete elimination the few *Borrelia* that migrate through the hemocoel toward the salivary glands could be possibly explained by an extremely fast movement of the motile spirochetes (Malawista and de Boisfleury Chevance, 2008; Dunham-Ems et al., 2009). Assuming that phagocytosis of *Borrelia* by tick hemocytes indeed reduces their number in the tick hemocoel and thereby negatively affects transmission of the spirochetes to the host via the salivary glands, RNAi-mediated silencing of *irc3-2* and *irc3-3* should result in a higher *Borrelia* number in tick salivary glands and subsequently in increased burden in murine tissues. Conversely, stimulation of phagocytosis by silencing of *irtep* should theoretically lead to reduced infections. However, the T-TEPs group-specific silencing in infected nymphs followed by their feeding on naïve mice did not confirm this view. RNAi-mediated silencing of *t-teps* did not result in any meaningful relationship with spirochete burden detected in ear biopsies during the early stages of infection. Only group silencing of *irc3-1,2,3* resulted in an apparently lower spirochete burden in the murine heart (Figure 7), however, more demanding transmission experiments should be performed either to reinforce or modify the statistical significance of this result for a reasonable interpretation.

Another scenario we can speculate about is that engulfment of spirochetes by tick hemocytes actually protects *Borrelia* against antimicrobial activity in tick plasma during their movement from the gut to the salivary glands. A “protective” role of tick hemocytes was proposed for the intracellular tick-borne pathogen, *A. phagocytophilum* (Liu et al., 2011). Secreted *I. scapularis* protein11 (P11) binds to bacteria and facilitates infection of tick hemocytes that serve as a vehicle for the internalized pathogen on its route toward the salivary glands. Silencing of P11 by RNAi or immunization of mice with anti-P11 antibodies significantly suppressed the *Anaplasma* burden in the tick hemolymph or salivary glands. The same effect was achieved by inhibition of *A. phagocytophilum* phagocytosis by injection of polystyrene beads into the *I. scapularis* hemocoel (Liu et al., 2011). Regarding the extracellular *Borrelia* spirochetes, the previous work by Johns et al. (2000, 2001) reported that *B. burgdorferi* inoculated into the *I. scapularis* hemocoel were eliminated much more slowly compared to the same experiment performed with the incompetent vector *D. variabilis*. Another *in vitro* study focused on phagocytosis of GFP-expressing *B. burgdorferi* by tick cell lines IDE12 and DAE15 derived from *I. scapularis* and *D. andersoni*, respectively, demonstrated that IDE12 cells required significantly more time to internalize and kill the spirochetes relative to DEA15 cells. Some intact coiled spirochetes (retaining GFP fluorescence) could be found in IDE12 cells as late as 7 days following their co-incubation (Mattila et al., 2007). However, our result showing that pre-injection of latex beads into infected nymphs had no apparent effect on *Borrelia* transmission (Figure 7B) suggests that tick hemocytes do not protect spirochetes on their route toward the salivary glands.

Certainly, many other factors might be affected by manipulating the tick complement-like immune responses, such as an impaired balance between *Borrelia* spirochetes and the commensal microflora (Narasimhan and Fikrig, 2015), which makes an unequivocal interpretation of our results more difficult. Therefore, more detailed investigations of tick-*Borrelia* inter-relationships in the midgut, hemocoel and salivary glands of refractory vs. competent tick species (Johns et al., 2001; Soares et al., 2006; Mattila et al., 2007) might shed more light which tissue plays the decisive role that determines the tick's capacity to act as a vector for Lyme disease.

## Author contributions

VU, OH, RS, PK conceived the study and designed experiments. VU, OH, HH, RS performed the experiments and analyzed data. VU, PK wrote the paper.

### Conflict of interest statement

The authors declare that the research was conducted in the absence of any commercial or financial relationships that could be construed as a potential conflict of interest.
