# Supplementary material for: Tick Thioester-Containing Proteins and Phagocytosis Do Not Affect Transmission of Borrelia afzelii from the Competent Vector Ixodes ricinus
Source: Front Cell Infect Microbiol. 2017 Mar 16;7:73. doi: 10.3389/fcimb.2017.00073 (PMC5352706; doi:10.3389/fcimb.2017.00073)
Supplement: Supplementary file 1 [file Table1.DOCX]

**Table S1│The number of *Borrelia* CB43^1^ in murine ears biopsies taken at one week intervals determined by qPCR.**

^1^  Number of spirochetes normalized to 10^5^ mice genomes
