# Supplementary material for: Tick Thioester-Containing Proteins and Phagocytosis Do Not Affect Transmission of Borrelia afzelii from the Competent Vector Ixodes ricinus
Source: Front Cell Infect Microbiol. 2017 Mar 16;7:73. doi: 10.3389/fcimb.2017.00073 (PMC5352706; doi:10.3389/fcimb.2017.00073)
Supplement: Supplementary file 2 [file Image1.PDF]

Supplemental Figure

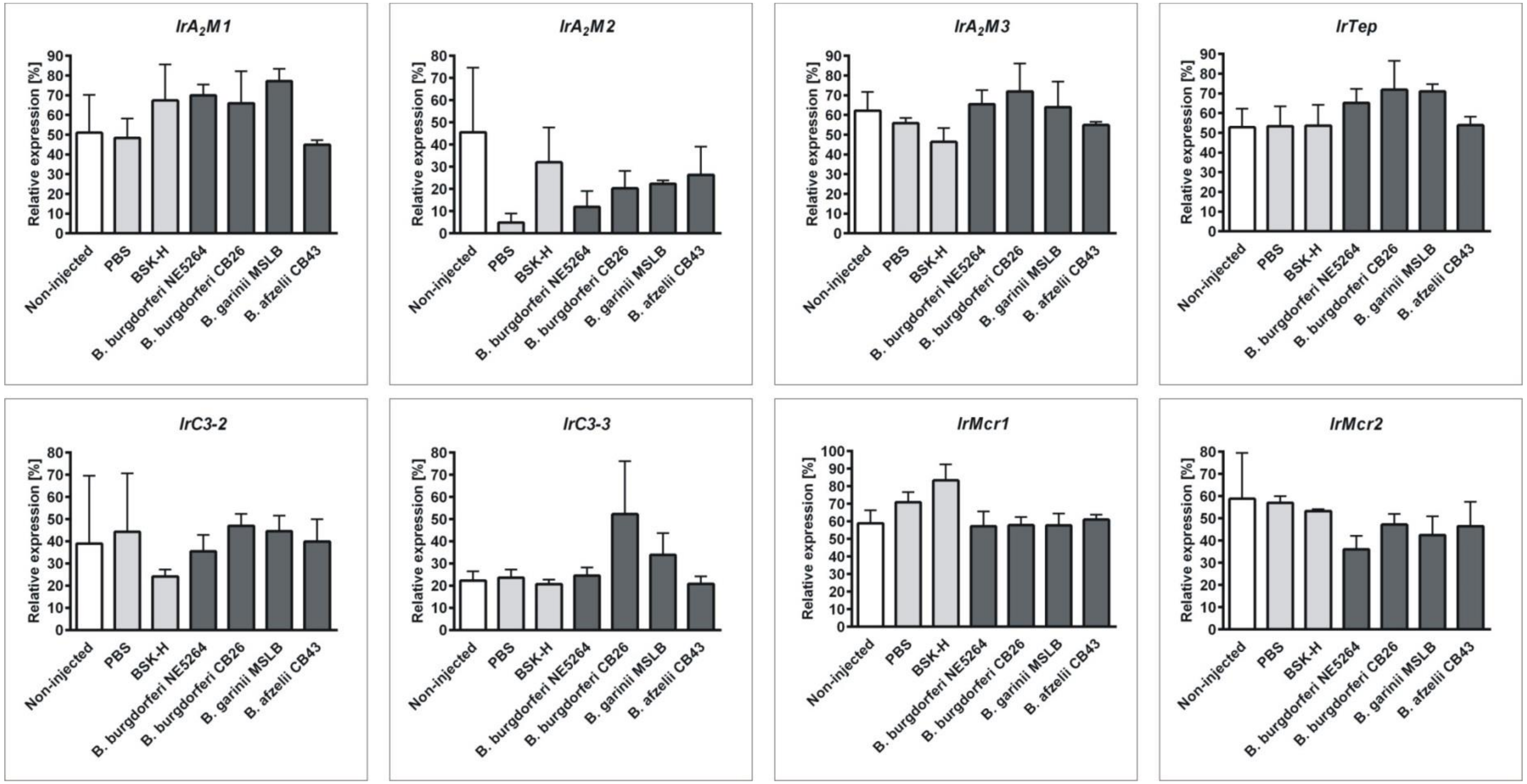

Figure S1 | Expression of *t-teps* in response to *Borrelia* sp. injection. Adult unfed *I. ricinus* females were injected with four different species of *Borrelia burgdorferi* s.l. complex (*B. burgdorferi* NE5264, *B. burgdorferi* CB26, *B. garinii* MSLB and *B. afzelii* CB43) or with sterile PBS, BSK-H medium as aseptic injection controls. Total RNA was isolated from the whole body homogenates, 12 h after the inoculation, and transcribed into cDNA. Expression is shown in relation to *elongation factor-1* as a housekeeping gene. Expression of *t-teps (*except *irc3-1,* see Figure 6) genes were not responsive to the *Borrelia* sp. injection. The error bars represent standard errors from three independent biological replicates.
